# Method of Directing Second Dentition

**Published:** 1854-10

**Authors:** James Taylor


### Dental Register of the West

VOL. VIII. OCTOBER, 1854. NO. 1.

### Original Communications.

### METHOD OF DIRECTING SECOND DENTITION.

BY JAMES TAYLOR, M.D., D.D.S.

Continued from page 215.

In our last we promised to continue this subject and give cases illustrating practice; we then condemned the practice of extracting the lateral incisors of the deciduous set to give room for the central incisors of the permanent. The extraction of the deciduous cuspid to give room for the permanent lateral is seldom if ever advisable. This practice is the cause of the numerous *tusks* which adorn the mouths of so many. It will be asked how then shall these teeth get room when they are coming in perfectly out of place—crowded and twisted? We answer that patience and faith in the natural operations of the economy will be generally sufficient, yet a misdirected tooth can be much relieved by proper pressure made in almost any manner by the parent or child itself, this pressure may be made with the parent's fingers and repeated daily; or, if an upper tooth is pointing inward and if left alone will strike inside of the inferior teeth, a small stick of wood placed on the under teeth projecting just far enough in the mouth, so that when the teeth are brought together the end of it may press on the inner or palital edge of the upper tooth, this when bitten upon presses the upper tooth out to its place and where the points do not strike on the occlusion of the teeth, will, if persevered in, often put in proper place the displaced tooth, and thus prevent the after necessity for a mechanical fixture for that purpose.

It should be borne in mind that a majority of teeth—I mean the front teeth—appear much out of place and as if, they would be very irregular when they first make their appearance and yet as the jaws expand and the teeth protrude they acquire room and take the right position. The solicitude of the parent, and the apparent irregularity observable by the Dentist, often urge something to be done, when *let alone* is all that is necessary.

Decay most usually takes place in the molars of the deciduous set, and thus one or more of these teeth are often lost by the time the incisors of the permanent set make their appearance. When this is the case, we find the crowded condition of the incisors is much sooner relieved, and thus that practice most in accordance with the condition and development of the teeth is pointed out. For, if any teeth are removed to give room, they should be back of the cuspids; and, besides, the organs of displacement here are smaller than the deciduous ones; and even should it occasion a crowded condition of the teeth at this part of the denture, it is not so observable, and the loss of a tooth to remedy irregularity here, would not marr the beauty of the denture. We would not, however, advise the injudicious extraction of even any of these teeth to relieve a crowded condition of the incisors, unless the crown of the tooth was well protruded and it was absolutely certain they could not make room for themselves. It may be asked, which one of these teeth (the deciduous molars) would we remove? If the teeth are all sound we should first see if either was being loosened, and if so, indicating its early loss, we should remove it. If all circumstances of this kind were absent, we should extract the first.

As before remarked, we believe in letting this operation of the economy go on in its most natural manner and hence extract as few of the deciduous teeth as possible, until they have been loosened or are in the way of the permanent ones which are to fill their place.

Children are very often brought into our office troubled with more or less pain in their molar teeth. If we find the front teeth are making sufficient room for themselves and the pain can be quieted by the application of some innocent preparation, such as Oil Cloves and Sulph. Ether, equal parts, or two parts Ether and one of Oil Cloves, then add as much Gum Champhor as they will dissolve. A drop of this on a little cotton applied to the aching tooth generally relieves. If, however, the incisors have not room, and these teeth are troublesome, we select the one which appears to be most affected and extract it, and if we should have to take out one on either side, we should feel that we were benefitting our patient, not merely in the present relief of pain, but in the arrangement of the permanent denture.

That practice which merely takes cognizance of one or two palpable difficulties, having no reference to collateral effects which must flow from it, is not much if any short of Quackery.

There is no part of the Dental practice requiring so profound a knowledge of the operations of the economy as that which relates to the eruption of the permanent teeth; a mere superficial observer will be constantly led astray, inflicting at almost every step far more injury than benefit. We are satisfied that our oldest and best practitioners are led by constant observation to feel that it is perhaps better not to do enough than too much. We are also satisfied that while there is no part of Dental practice which requires more skill and experience, there is also no part more trying to the feelings of the humane operator. It is only a sense of duty which would induce us to inflict pain, yet we should feel that duty is inexorable and demands decided action. A firm and decided course is not inconsistent with great tenderness; and we are satisfied that the child will sooner, and with far less trouble, yield to the proper treatment by a kind manner which swerves not from the path of duty, than from any other. Children should not be deceived, and the disposition we so often see in parents to induce a compliance in their children to submit to have a tooth or two extracted, by telling them it will not hurt, should be properly reproved; get the child's confidence by telling the truth, and future trouble is nearly at an end.

The first and most common irregularity of the teeth which demands special treatment to remedy, is the front or lateral incisors, one or more striking inside of the lower teeth at every occlusion of the jaws.

This is not only the most frequent irregularity which presents itself, but it is also one of the most easy to relieve. This condition of the teeth can be very often prevented by the use of the stick, or pressure made by the parent, repeated very often every day, before the tooth has so far protruded through the gum as to really strike the lower teeth. When, however, this is the case, every occlusion of the mouth would tend to keep up the irregularity. The object now is to alter this unnatural strike of the teeth, and so long as the other teeth are permitted to come together, this cannot be accomplished.

The most simple and effective contrivance to remedy this defect is a cap for three or four of the under teeth. This we obtain by first taking an impression of these teeth in wax, then secure a plaster model and next a metallic one, we then take a soft and good piece of gold plate about the thickness of that used for upper plates for full sets. This is struck up to neatly fit these teeth, we then punch two holes opposite two of these teeth each on the palital part of this plate, then notch the plate on the labial part. The plate is then adjusted to fit the teeth and next an inclined plane is soldered o this gold cap; this inclined plane is so attached that when the cap is set on the lower teeth and the mouth closed, the inclined plane strikes the palital face of the upper tooth or teeth that incline too much in.

It is now ready for fastening on, and this is effected by passing two thread ligatures through the holes punched on the palital portion of the plate or cap. It is now ready for adjusting to the teeth, and the ligatures are passed between the teeth so that each ligature shall pass around or between the teeth, and the cap forced on the teeth. The ends of the ligatures are then tied over the notched plate on the labial face of the teeth, and thus secure the fixture to its place. This cap and inclined plane keeps the teeth apart and at every occlusion of the teeth, the inclined plane strikes the palital face of the irregular teeth or tooth and forces it out to its proper place. If the adjoining teeth overlap the misdirected teeth, we pass a doubled waxed thread between the teeth and under the irregular teeth and over the overlapping teeth and tie it fast. This ligature keeps up a tension on the irregular tooth, drawing it out to its place and at the same time makes room for its position in the arch, by wedging, as it were, the overlapping teeth apart, acting indeed just as wedges on either side. Should this ligature be disposed to slip too high up on the neck of the teeth ; it may be tied down by another ligature passed between the teeth and tied with the knot at the cutting and approximal edges of the teeth, or by the adjustment of one or more small gold hooks applied to the incisor. These hooks are bent over the points of the teeth and the upper end either notched or bent out to catch and hold the ligature.

These fixtures are all that is generally necessary to put into place teeth of this character. And when this has been worn until the teeth strike fairly on the outer edge of the lower teeth, it can be removed, for then the continued closing of the teeth together will of itself soon finish the operation.

We have had a few cases in advanced life, where we have resorted to the following contrivance; make a stiff and flattened bar of gold to fit the labial face of the incisors and canine teeth, standing off from the tooth which is within the arch. We solder two hooks to this bar which catch over the points of two of the incisors and keeps the bar off from the gums. We then pass a ligature around the neck of the irregular tooth and tie it fast to the bar. This very soon draws the tooth to its proper place.

Should the irregular tooth strike inside of the under teeth, a cap with an inclined plane attached, would still be necessary to hold the teeth apart so that the tooth can pass out into its proper place in the circle. We have now given the most simple cases of marked irregularity, requiring mechanical fixtures for regulation, and as we regard, the most simple means that can be used for remedying the irregularity. We do not pretend that there is anything new in principle about the plan of treatment here laid down. The principle is the same as that recommended by Catalan and others, and the inclined plane the same, substituting however a mere cap of gold to three or four teeth, when but one tooth or two are to be acted upon, for the extended gold bar. Our plan is indeed more like that recommended by Prof. Harris, only we do not think it necessary that our cap shall encase so many teeth. We are in the habit of applying this fixture very early in life, earlier indeed than any other mechanical contrivance we can use. For so soon as the inferior incisors of the permanent set are in place or fully developed through the gum, the cap can be attached to them, and this indeed is the period of life when most frequently called on to use this appliance. For they are not more than fairly through and perfectly developed before the upper ones are making their way into a proper articulation, and should this articulation be wrong every additional tooth obtained renders the difficulty more and more serious.

There are many cases of irregularity. Where the teeth, especially in the upper jaw, are overlapping each other and present a very ugly appearance, when the ligatures as applied by Delabarre is all that is sufficient, and unless some of the teeth strike inside of the under ones, no other appliance is necessary to accomplish the object in view. We give the following case as illustrative of this:

The two central incisors are prominent, and project. The lateral incisors are overlapped by the central and cuspidati, so that about one half of the laterals are in view. The first bicuspids are within the proper circle. All the teeth of the permanent set are thıough, and yet none really strike inside of the lower teeth, and yet the cusps of the lower and upper teeth do not properly articulate, but glance on their points, and do not pass into their appropriate depressions. Now, we regard the careful observation of this point as of great consequence, and one that should be kept constantly in view in all our appliances for regulating deformity.

The case we have referred to was one that had withstood all the efforts of an old practitioner for more than a year, and, indeed, had passed through two hands. The extraction of the first bicuspids was at length advised, and even the separating of the front and lateral incrsors. To avoid this, we were called on, and after a thorough examination of the case, and measurement of the circle in which the teeth should stand, we saw that all the space necessary could be thus obtained. The individual was in his eighteenth year of age, teeth all sound. Our ligatures throughout the entire case were stroug patent thread, made double and waxed. This was passed first between the anterior and posterior molars, and brought forward around the anterior molar, and secured with a knot, then brought forward over the posterior bicuspid, then both ends passed between the bicuspids, and carried around the lingual neck of the anterior bicuspid, which stood too far in. Then between this tooth and the cuspid, then over the labial neck of this tooth, and then between this and the lateral incisor, which was also too far in and around the lingual neck of this, and between this and the front incisor. The same operation was gone through with on the other side. Thus the two ligatures used met at the front incisor teeth, and after being drawn tight, were tied over the labial face of these teeth. It will be observed that the thread which passed between the teeth, was in four strands, making what answered as a good wedge for these teeth, for we took good care to tie it down on the crowns of the teeth by a double ligature passed between the teeth and above the main ligatures and tied at the points of the teeth. This at first was an easy matter, as the teeth was so close that the knot would not slip up or allow the main ligature to do so. Towards the close of the operation we secured it down by hooks, catching over the points of two or three teeth. In this plan we have the traction exerted at the same time that the wedges are making room, and in some four or five weeks the teeth all came into their proper place and were secured by single ligatures loosely applied until they became firmly fixed by the adaptation of the alveolus around them.

We would here remark for the information of the young Practitioner, that for the first two or three days, all appliances which require much force exerted to bring the teeth in place, produce come considerable soreness of the teeth, but after this period is passed the soreness is less, although the teeth often appear somewhat loose. Care should, however, be taken, not to apply the fixtures so tight as to produce inflammation in the alveolo-dental membranes. We sometimes merely retain the position gained for a few days until all soreness has left and then tighten the ligatures again. The ligatures and fixtures should be taken off every two or three days, and before re-applying, the teeth carefully cleaned, using daily some good astringent wash to keep down irritation of the *gums.* The same treatment here recommended, it will be seen, is applicable in either the upper or lower jaw, when the teeth in their irregularity do not really articulate adversely.

Before closing this article for the present number of the Register, we will give our method of bringing into place an oblique upper incisor. It will be seen that it differs but very little from that recommended by Dr. Evans, of Paris, or that which he recommends for cases not refractory. The general plan we believe is the same as is generally pursued by the profession, only differing a little in detail.

After having acquired space by wedging the contiguous teeth apart, to get room, we adjust a band of gold, made of 22 carrot plate, around the crown of the oblique tooth. This band should be adjusted as near the gum as possible, yet embracing the crown so as to retain its position. After it has been well adapted to the tooth, so that it will not slide or turn on the tooth, the ends should be soldered together. We then take a bar or wire of 18 carrot gold, and long enough to reach from this tooth back to the molars. We then solder one end of this to the band, at a point where the lingual face of the teeth should be, and at an angle throwing the bar from the teeth. The bar is now hammered and burnished to give it elasticity. It is then adjusted to the tooth and the bar drawn to the molar tooth and fastened by a ligature. The bar can be at all times so bent with a pair of pliers, as to give merely the amount of traction desired. If necessary, as the tooth turns, the bar may be cut off, and resoldered.

We had intended giving in this number, a few cases of irregularity, relieved by a bar and ligatures, but the wood cuts are not ready. They will appear in the next.

